# Special Issue “Researching Sports Biomechanics for Disabled People”

**DOI:** 10.3390/sports9120161

**Published:** 2021-11-26

**Authors:** Luca Paolo Ardigò, Ibrahim Ouergui, Johnny Padulo, Hadi Nobari, Damiano Formenti

**Affiliations:** 1 Department of Neurosciences, Biomedicine and Movement Sciences, School of Exercise and Sport Science, University of Verona, Via Felice Casorati 43, 37131 Verona, Italy; 2 High Institute of Sport and Physical Education of Kef, University of Jendouba, Boulifa University Campus, Kef 7100, Tunisia; ouergui.brahim@yahoo.fr; 3 Department of Biomedical Sciences for Health (SCIBIS), Università degli Studi di Milano, Via Giuseppe Colombo 71, 20133 Milano, Italy; johnny.padulo@unimi.it; 4 HEME Research Group, Faculty of Sport Sciences, University of Extremadura, 10003 Cáceres, Spain; hadi.nobari1@gmail.com; 5 Department of Physical Education and Sports, University of Granada, 18010 Granada, Spain; 6 Department of Exercise Physiology, Faculty of Educational Sciences and Psychology, University of Mohaghegh Ardabili, Ardabil 56199-11367, Iran; 7 Sports Scientist, Sepahan Football Club, Isfahan 81887-78473, Iran; 8 Department of Biotechnology and Life Sciences, University of Insubria, Via J-H Dunant 3, 21100 Varese, Italy; damiano.formenti@uninsubria.it

Disabled people compete at high levels in several sport disciplines and physical activity for this population has become a high interest area of study in biomechanics. However, the traditional research approach in biomechanics needs to be adapted to cope with the unique requirements of disabled people. To help them with their functional disabilities, researchers need to re-think their methods of investigation. Paralympic athletes compete in both individual and team sports, locomotive and non-locomotive disciplines, short-lasting high-intensity and long-lasting low-intensity efforts, etc. Sport is acknowledged as being of immense value to sedentary disabled people as well. As is already the case with able-bodied people, sports science is in part required to support performance improvement. This applies to both training and racing among other sport disciplines. This Special Issue includes eight articles (six original and two reviews) published belonging to three research areas: (1) testing, (2) training and (3) both testing and training.

Regarding testing, Kelli et al. assessed (including reliability) one trunk stabilizing muscle (transverse abdominal) activity—in terms of thickness—at rest and during exercise in post-stroke hemiparetic individuals [1]. Compared with controls, authors showed lower thickness and higher non-paretic vs. paretic asymmetry in patients. Performance, mechanical output and metabolic expenditure measurement were investigated in tetraplegic and paraplegic handbike athletes riding at submaximal speeds by Fischer et al. [2]. Compared with paraplegic athletes, tetraplegics were featured by lower speed, lower mechanical power and lower overall mechanical efficiency but the same metabolic cost. Reina et al. assessed kinematics and dynamics variables in adult football players with cerebral palsy performing counter movement jumps (CMJ) with arms swing and with or without headers [3]. Compared with header-less, authors showed more extended posture during landing and lower overall inter-leg asymmetry while jumping with header. Finally, different physical fitness tests (maximal handgrip isometric force, CMJ, throwing speed [TS] and Yo-Yo intermittent recovery test level 1 [Yo-Yo IR1]) performances and body composition and their mutual relationships were investigated in elite goalball players by Goulart-Siqueira et al. [4]. Moderate-to-high correlations were found among most performances, as well as correlation between body fat percentage and both CMJ and Yo-Yo IR1 results in addition to CMJ’s highest correlation with TS.

Regarding training, Aidar et al. assessed the effects of one-session variable-resistance (elastic bands) strength training in Paralympic powerlifters [5]. Compared with traditional strength training, authors did not find any differences in force indicators but (higher) fatigue and (longer) time to reach maximum isometric force. The outcomes of a ten-week home-based exercise program’s effects were investigated in elite wheelchair basketball players by García-Gómez et al. [6]. Compared with controls, the exercise group was featured by increased shoulder extension range of movement but no pain change either in players previously reporting it nor in those free of injury. Then, Jacinto et al. performed a systematic review of studies on strength training and its effects in individuals with intellectual disability [7]. Authors highlighted increments in strength, balance and fat-free mass and decrements in fat mass and waist circumference.

Regarding both testing and training, a narrative review of studies on biomechanics’ contributions to training, equipment and performance in Paralympic athletes was published by Fletcher et al. [8]. They focused their review on the different sport-specific body postures (standing, sitting and horizontal) to be taken into account and on the need for tuning running/jumping-specific prostheses and wheelchairs/handbikes/watercraft (shell, canoe or kayak) designs to each athlete.

In conclusion, the published works demonstrate that the study of biomechanics of disabled people’s sport and physical activity has still considerable room for improvement from a scientific viewpoint. A biomechanically sound training plan is shown to be valuable to maintain health and prevent injuries in disabled people. This Special Issue represents a useful guideline for physical exercise professionals to design and adjust training plans not only to improve physical fitness but even to improve quality of life of disabled people. Overall, published works’ authors provide practitioners dealing with sports for disabled people with specific hints on disability and sport-specific testing and training options to optimize performance making wise use of current biomechanics knowledge.
